# Genome Sequences of Barmah Forest Virus Strains Isolated from Mosquitoes Trapped in Australian Defence Force Training Areas Reveal Multiple Nucleotide Insertions in the 3′ Untranslated Region

**DOI:** 10.1128/MRA.00969-19

**Published:** 2019-10-10

**Authors:** Wenjun Liu, Joanne R. Kizu, Christina Hoare, Ian R. Mitchell, Penelope J. Gauci, Aneta J. Gubala

**Affiliations:** a Arbovirology Department, Australian Defence Force Malaria and Infectious Disease Institute, Enoggera, Queensland, Australia; b Land Division, Defence Science and Technology Group, Fishermans Bend, Victoria, Australia; KU Leuven

## Abstract

The complete genome sequences of two Barmah Forest virus (BFV) strains isolated from mosquitoes trapped in the Australian Defence Force (ADF) training areas during 2017 and 2018 reveal multiple nucleotide insertions in the 3′ untranslated region (UTR) of ADF BFV strains compared with the BFV prototype strain whole-genome sequence in GenBank.

## ANNOUNCEMENT

Epidemic polyarthritis (EPA) caused by Barmah Forest virus (BFV) infection is the second most frequently notified arboviral disease in Australia after Ross River virus (RRV), with approximately 1,200 cases reported annually over the last decade (1). BFV is a positive-sense, single-stranded, enveloped RNA virus in the Alphavirus genus of the Togaviridae family (2), and it is an endemic and enzootic virus in Australia that has a natural animal-mosquito-animal transmission cycle. Native animals, such as wallabies and kangaroos, are thought to be the main animals involved in the transmission cycle of infection (2). Several mosquito species, including Culex annulirostris, Aedes vigilax, Aedes normanensis, Aedes notoscriptus, and Verrallina funerea, were recorded to carry the virus (2, 3). Humans can be infected by spillover of virus, resulting in disease that is similar to but milder than RRV infection, having relatively benign symptoms such as fever, rash, arthralgia, and myalgia (2–5). There is no specific antiviral treatment, and no commercial BFV vaccine is available (2, 6, 7).

Full-genome sequences allow for intensive molecular evolutionary studies on vaccine development and disease control measures. Prior to our study, there was only one complete BFV genome sequence available in GenBank, that of the prototype strain BH2193, which was isolated from C. annulirostris mosquitoes trapped in the Barmah Forest area of Northern Victoria in 1974 (8).

We present here two BFV genome sequences obtained from the Australian Defence Force (ADF). The MIDITullyA.2017 strain was isolated from a homogenized pool of 20 Verrallina sp. mosquitoes captured in the ADF Tully training area (TA) (17.9°S, 145.9°E; Queensland, Australia) in 2017, while the MIDIWBTA.2018 strain was isolated from a homogenized pool of 20 *C. annulirostris* mosquitoes captured in the ADF Wide Bay TA (25.3°S, 152.8°E; Queensland, Australia) in 2018. The trapped mosquitoes were sorted according to species, placed into a 2-ml screw-cap vial with 1 ml MD (2% fetal bovine serum in RPMI 1640, 50 µg/ml penicillin/streptomycin, 50 µg/ml gentamicin, 2.5 µg/ml amphotericin B, and 10 mM HEPES) and 4 or 5 zirconium silica beads, and shaken for 1 min 30 s in a chilled block using a MiniBeadbeater-96 sample homogenizer (BioSpec Products, Bartlesville, OK, USA), followed by centrifugation (twice at 17,000 × *g* for 10 min at 4°C, with tube rotation). The mosquito homogenates were used to infect C6-36 Aedes albopictus cells for 3 days at 30°C as previously reported (9). Culture supernatant was harvested, and cell debris was removed by centrifugation at 5,000 × *g* for 10 min. The resulting supernatant was stored at −80°C and employed as a source of virus. Total viral RNA was extracted from a 140-μl tissue culture supernatant using an RNeasy minikit (Qiagen) prior to being converted to cDNA using the Repli-g whole-transcriptome amplification single-cell kit (Qiagen). The cDNA library for each virus was prepared individually using the Nextera XT kit and sequenced on a MiSeq instrument using a Reagent Micro kit version 2 (300 cycles; Illumina) according to the standard protocol. The MiSeq sequence data were assembled by mapping the reads to a reference genome, that of BFV prototype strain BH2193 (GenBank accession number NC_001786) using Geneious software R11 (version 11.1.2). The nucleotide sequences in the 3′ untranslated region (UTR) of both ADF strains were confirmed by Sanger sequencing of the reverse transcription and PCR amplicons using 5′/3′ rapid amplification of cDNA ends (RACE) kit (Roche, Germany) according to the manufacturer’s instructions, as described previously (9). The 3′ UTR Sanger sequence results were trimmed and mapped to the BH2193 reference strain with overlapping to the E1 protein 3′ nucleotide sequence end using Geneious software. The MiSeq sequence data were remapped to the generated 3′ UTR sequences of both ADF strains to confirm the sequences. The open reading frames and annotations were determined by mapping each consensus sequence to the BH2193 genome using Geneious. The complete BFV sequences were aligned using ClustalW for calculating the nucleotide and the deduced amino acid differences.

In total, 1,064,168 and 774,260 paired-end reads (2 × 150 nucleotides [nt]) were sequenced for MIDITullyA.2017 and MIDIWBTA.2018, with 706,386 and 400,471 reads mapping to BFV, respectively. The resulting consensus sequence revealed that MIDITullyA.2017 and MIDIWBTA.2018 have genome lengths of 11,574 nt and 11,563 nt [without 3′ poly(A)], and G+C contents of 48.5% and 48.6%, respectively. At the nucleotide level, both ADF BFV strains shared 97.2 to 97.3% similarity with the prototype BH2193 strain (Table 1). However, the similarities of the 3′ UTRs are only 78.4% and 80.68%, respectively, in pairwise comparison with the prototype strain. The 99.5% nucleotide sequence similarity between MIDITullyA.2017 and MIDIWBTA.2018 isolates over a 2-year period from two different ADF training sites indicates that this virus strain is currently endemic in ADF training areas of Queensland, Australia (Table 1).

**TABLE 1:** Nucleotide and amino acid sequence pairwise comparisons of ADF BFV strains with the prototype BH2193 strain

Strains compared	Similarity (%)						
	Genome	5′ UTR	Nonstructural genes		Structural genes		3′ UTR
			nt	aa	nt	aa	
BH2193 and MIDITullyA	97.2	95.20	98.1	99.0	98.4	99.2	80.68
BH2193 and MIDIWBTA	97.3	96.82	98.0	99.0	98.2	99.1	78.40
MIDITullyA and MIDIWBTA	99.5	98.40	99.7	99.8	99.7	99.7	97.20

The deduced amino acid sequences of the open reading frames of both ADF BFV strains contain an additional stop codon between nonstructural nsP3 and nsP4 proteins, as reported in other alphaviruses (10, 11). In comparison with BH2193, MIDITullyA.2017 and MIDIWBTA.2018 have a total of 32 and 33 amino acid (aa) substitutions, respectively, evenly spread in nonstructural and structural proteins. It is not clear if these deviations from the prototypic sequence affect viral fitness and disease transmission and warrant further investigation.

**FIG 1:**
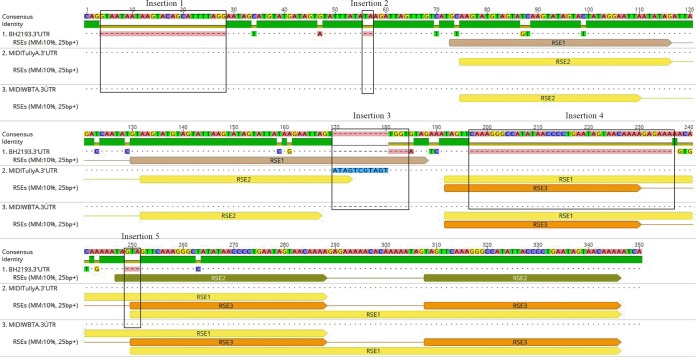
Nucleotide alignment of 350-nt 3′ UTR sequences of two ADF BFV strains with that of the prototype BH2193 strain. The repeat sequence elements (RSEs) were determined using Geneious software (version 11.1.2) with a minimum repeat length of 25 bp, a maximum mismatch of 10%, and an excluding repeat up to 10 bp longer than contained repeat. The dots indicate the consensus sequence of three BFVs, while letters in individual sequences indicate nucleotide substitutions. Dashes indicate insertions/deletions. The RSEs were annotated.

Nucleotide alignment of the 3′ UTR sequence of two ADF BFV strains with that of the prototypic strain revealed multiple insertions in the 3′ UTR of both ADF strains. These insertions in both ADF strains are similar, except for insertion 3 of MIDITullyA.2017, which is 4 nt longer than that of the MIDIWBTA.2018 strain. These insertions disrupted the repeat sequence elements (RSE) (12) in the 3′ UTR that were originally identified in the prototype BH2193 strain, and new RSEs are formed in both ADF strains (Fig. 1). These insertions were confirmed by Sanger sequencing of reverse transcription and PCR amplicons of the 3′ UTR of both ADF strains. The impact of these insertions on BFV replication and transmission is unclear. Previous studies of other alphaviral genomes have shown that deletions of RSEs in the 3′ UTR region have significant effects on viral gene expression, replication, and protein translation in mosquito and avian cells, possibly through interaction with cellular proteins and microRNAs (miRNAs) (13).

### Data availability.

Raw next-generation sequencing (NGS) reads were deposited in the Sequence Read Archive under the accession numbers SAMN11130004 and SAMN11130005 and BioProject number PRJNA527173. The BFV genome sequences in this communication are publicly available in GenBank under the accession numbers MN064696 and MN064697.
